# Ecological and socio-demographic differences in maternal care services in Nepal

**DOI:** 10.7717/peerj.1215

**Published:** 2015-09-03

**Authors:** Vrijesh Tripathi, Rajvir Singh

**Affiliations:** 1 The Faculty of Science and Technology, The University of the West Indies, St Augustine Campus, Trinidad & Tobago, West Indies; 2 Cardiology Research Centre, Heart Hospital, Hamad Medical Corporation (HMC), Doha, Qatar

**Keywords:** Nepal Demographic Health Survey, ANC, Safe delivery, Ecological zones

## Abstract

**Background.** Nepal is set to achieve MDG-5 goals by end of 2015. However, maternal health parameters will remain way below those of developed countries. This study was conducted to assess the factors contributing to utilization of ANC and safe delivery services with the aim of furthering overall maternal health parameters in Nepal.

**Material and Methods.** Using survey data from the Nepal Demographic and Health Survey 2011, socio-economic and demographic factors associated with the utilization of ANC and safe delivery services among women aged 15–49 years who gave births during the last three years preceding the survey are examined. Data was segregated into three ecological zones: Mountain, Hill and Terai zones for univariate analyses. Data from all three zones was then pooled for univariate and multivariate logistic regression analyses of Antenatal Care and Safe Delivery services in Nepal.

**Results and Conclusion.** The analyses show that rural place of residence is at a disadvantage in receiving ANC (OR, 0.8; 95% CI [0.7–0.9]) and ensuring safe delivery (OR, 0.6; 95% CI [0.5–0.7]). Woman’s education, husband’s education and wealth quintile are significant factors in ensuring ANC and safe delivery services. Further, the analyses show that Budh/Muslim/Kirat/Christians are at a significant disadvantage in ensuring safe delivery (OR, 0.8; 95% CI [0.7–0.9]) as compared with Hindus. Though ecological zones lost their significance in receiving ANC, women in the Terai region are at a significant advantage in ensuring safe delivery (OR, 1.7; 95% CI [1.2–2.1]).

**Recommendation.** Segregated targets should be set for the different ecological zones for further improvement in maternal mortality rates in Nepal.

## Introduction

Almost 300,000 women die every year from causes related to pregnancy and childbirth around the world. Maternal deaths mainly occur due to obstructive haemorrhage during or just after delivery, sepsis, and complications of unsafe abortion, malaria, and HIV. These deaths are avoidable by presence and attendance of trained health care workers, before, during and after delivery for intervention and management of complications (UN, 2011; Imeda, 2013). At the level of policy implementation, this translates into offering effective antenatal care (ANC) services and ensuring a skilled birth attendant (SBA) during childbirth for safe delivery.

Nepal is one of the few countries set to achieve the United Nations defined Millennium Development Goals (MDGs) by reducing under-five mortality rate (U5MR) from 158 (per 1,000 live births) in 1990 to 54.4 (per 1,000 live births) in 2011 (MoHP, New ERA, and ICF International Inc, 2012) and reducing maternal mortality ratio by three quarters from 850 to 213 per 100,000 live births by 2015 (UN, 2011; Pant et al., 2008). However, the larger goal is to make reproductive health care accessible to all. The MDG-5 has set the goal of increasing the proportion of births attended by a SBA to 60% in 2015. They have also set the target of ensuring at least four antenatal care visits during pregnancy to 80% in 2015. Though it seems likely that Nepal will achieve the projected target of 60% of childbirths having a SBA, Nepal Demographic and Health Survey 2011 (NDHS 2011) data suggests that that alone may not be enough to bring down maternal mortality ratio. Also, there is the fact that though the MDG-5 may be achieved, maternal mortality would remain a major public health problem in Nepal.

Nepal is a country in the Himalayas divided into three ecological zones: Mountain, Hill and Terai. It is important to focus on ecological differences in service delivery to develop an action plan for the future. Furthermore, it is important to note that 90% of Nepal’s population lives in rural areas where ANC services and safe delivery figures are not very encouraging. The NDHS 2001 found that around 18% of women in urban areas did not receive any ANC compared with 53% of women in rural areas. 44% of women in the Terai, 56% of women in the Hills and 69% of women in the Mountains went without any ANC (MoHP, New ERA and ORC Macro, 2002). Since socio-economic and demographic factors are the core of population-based surveys, this paper examines the influence of socio-economic and demographic factors in the use of maternity care services across the three ecological zones in Nepal based upon data collected from the NDHS 2011. The paper analyses two important components of maternity health care, ANC and safe delivery services.

## Methods

The study uses raw data from nationally representative samples of ever-married women aged 15–49 years in NDHS 2011 conducted from February 2011 to June 2011. The response rate was 98% and the total number of women participants was 12,918 with 26,615 births (MoHP, New ERA, and ICF International Inc, 2012; Sreeramareddy, Harsha Kumar & Sathian, 2013). Those currently married women interviewed about births carried in the past three years totals 7,069. There were 1,516 births in the Mountain, 2,872 births in the Hill and 2,681 births in the Terai zones that were eligible for the study. The study examines socio-economic and demographic differences in the utilization of maternity care services across these three ecological zones.

### Ethics statement

The Nepal Demographic and Health Survey was conducted on an independently approved ethics review of the NDHS protocol by New ERA (non-governmental organisation) and ORC Macro International. This study is based on the NDHS data, which is available in the public domain with no identifiable information on the survey participants; therefore, this work is exempted from ethical review.

### Outcome events

ANC services include those women who had at least three antenatal care visits or at least two tetanus toxoid injections during pregnancy or one tetanus toxoid injection in pregnancy and at least one tetanus toxoid injection in the preceding three years and received iron and folic acid tablets for 90 or more days (Paul et al., 2011; Singh et al., 2012). Safe delivery is described as delivery attended by doctor/Auxiliary Nurses Midwife (ANM) or Lady Health Visitor (LHV) or health assistant/auxiliary health worker (AHW) or mother and child health workers (MCHW) or village health workers (VHW) or health professional or traditional birth attendant (TBA) or facility staff and health volunteers (FCHV).

### Covariates

Data was segregated according to the three ecological zones in Nepal, namely, Mountain, Hill and Terai. There is merit in segregating data for it allows a focus on aspects of ANC and Safe Delivery that may remain hidden in national level indicators (Katti, 1987). It may be easy to conjecture that terrain marks a difficulty in access to health facilities and the number of times a SBA can visit these women.

The key socio-demographic factors investigated include age, place of residence, education of self and husband, religion, occupation of self and husband, wealth quintile, frequency of listening to radio/reading newspaper and magazines, birth order and child status at birth. While age is a continuous variable, it is categorized into age groups 15–19 years, 20–24 years, 25–29 years and above 30 years to identify the age group that utilizes the ANC services the least. Place of residence is categorised as rural or urban. Education is categorised as no formal education, up to primary level, up to secondary level and higher. Religion is categorised as Hindu and Budh/Muslim/Kirat/Christians. Occupation is categorised as not working, unskilled or skilled work, and agriculture. The economic status of women is assessed computing a composite wealth index created using principal component analysis of household items related to possession of durable assets, access to utilities and infrastructure, and housing characteristics. Each woman is ranked based on a household asset score and is assigned to wealth quintiles. Accordingly, the first quintile was poorest 20% of the households and the fifth quintile was the wealthiest 20% of the households. A detailed description on the methodology adopted to construct the wealth index in NDHS dataset is provided in the NDHS 2011 national report (MoHP, New ERA, and ICF International Inc, 2012). This study combines the poorer and poorest and richer and richest in order to decrease the categories. In other words, we have three categories, poor, middle and rich representative of total population. Public messaging is important and is often the source of getting information for the woman. Hence, frequency of listening to the radio/reading newspaper and magazines is included to gauge the woman’s level of perception regarding family health initiatives.

### Statistical technique

Data are screened from Demographic and Health Survey website with due permission for analyses. Frequency with percentages is calculated for predictors and outcome variables. To examine the bivariate relationships of women’s social and demographic variables and healthcare outcomes data is stratified according to ecological zones and the dependent variables are ANC and safe delivery services. Chi-square tests are performed to see associations and univariate logistic regression analyses to know about odds ratios and 95% CI for all predictors. The data is pooled together because zone-wise bifurcation of data was not adequate for multivariate analyses. Univariate and multivariate logistic regression analyses are performed to assess the influential predictors for ANC and safe delivery services. Odds Ratios (OR) with 95% confidence intervals are presented in the tables (Harrell, 2001; Kleinbaum et al., 1998; Bewick, Cheek & Ball, 2005).

The C-statistics and Area under the Receiver Operating Characteristic (AUROC) curve are performed to see how well the predicted probabilities fit the developed models. The area under the receiver operating characteristic curve measures the ability of the developed models to discriminate utilization of ANC and safe delivery services. *P*-value 0.05 (two tailed) is considered for statistical significant level (Zou, O’Malley & Mauri, 2007). SPSS 21.0 statistical package is used for the analyses (IBM SPSS, 2012).

## Results

### Antenatal care services across the ecological zones

The number of women who received at least 1 ANC visit is 2,411, at least 2 visits are 2,251 and at least 3 visits are 1,961. Table 1 presents the results of univariate analyses of socio-demographic characteristics of 2,795 births that received ANC services according to ecological zones. These births included 537 births in the Mountain zone, 1,121 births in the Hill zone and 1,137 births in the Terai zone. The table shows that as the age of the woman increases, the utilization of ANC decreases in the Mountain, Hill and Terai zones (*p* = 0.001). ANC services are utilized by only 5% women above 30 years of age as compared with those in the 15–19 age groups (OR, 0.05; 95% CI [0.03–0.1]). Fewer women utilize ANC services in the rural as compared to urban place of residence in the Mountain (OR, 0.5; 95% CI [0.3–0.8]), Hill (OR. 0.5; 95% CI [0.4–0.6]) and Terai (OR, 0.8; 95% CI [0.2–1.0]) zones.

**Table 1:** Socio-economic and demographic characteristics and univariate analyses of ANC services according to ecological zones in Nepal, NDHS-2011.

	Mountain zone			Hill zone			Terai zone		
Variable	ANC 537(%)	Total 1,516	OR & 95% CI	ANC 1,121(%)	Total 2,872	OR & 95% CI	ANC 1,137(%)	Total 2,681	OR & 95% CI
**Age**									
15–19 yrs	56(83.6)	67	1.0	98(87.5)	112	1.0	137(87.8)	156	1.0
20–24 yrs	198(58.2)	340	0.3(0.1–0.5)	406(59.9)	678	0.2(0.1–0.4)	417(57.4)	726	0.2(0.1–0.3)
25–29 yrs	153(34.1)	449	0.1(0.05–0.2)	337(40.2)	838	0.2(0.05–0.2)	364(40.1)	908	0.1(0.06–0.2)
30+ yrs	130(19.7)	660	0.05(0.03–0.1)	280(22.5)	1244	0.04(0.02–0.1)	219(24.6)	891	0.04(0.03–0.06)
**P value**	**0.001**			**0.001**			**0.001**		
**Place of residence**									
Urban	38(52.8)	72	1.0	226(54.1)	419	1.0	332(45.9)	723	1.0
Rural	499(34.6)	1,444	0.5(0.3–0.8)	895(36.5)	2,453	0.5(0.4–0.6)	805(41.1)	1,958	0.8(0.2–1.0)
**P value**	**0.002**			**0.001**			**0.03**		
**Woman’s education**									
No formal education	274(27.3)	1,004	1.0	419(27.4)	1,530	1.0	479(31.8)	1,506	1.0
Primary	110(44.9)	245	2.1(1.6–2.7)	233(42.1)	554	1.9(1.6–2.4)	213(48.6)	438	2(1.6–2.5)
Secondary & above	153(57.3)	267	3.6(2.7–4.7)	469(59.5)	788	3.9(3.3–4.7)	445(60.4)	737	3.3(2.7–3.9)
**P value**	**0.001**			**0.001**			**0.001**		
**Husband’s education**									
No formal education	100(23.8)	420	1.0	180(24.5)	734	1.0	234(29.1)	804	1.0
Primary	150(30.0)	500	1.4(1.0–1.8)	271(34.0)	797	1.6(1.3–2.0)	251(40.1)	626	1.6(1.3–2.0)
Secondary	227(47.5)	478	2.9(2.2–3.9)	522(47.9)	1,089	2.8(2.3–3.5)	510(49.4)	1,032	2.4(2.0–2.9)
Higher	60(50.8)	118	3.3(2.2–5.1)	148(58.7)	252	4.4(3.2–5.9)	142(64.8)	219	4.5(3.3–6.2)
**P value**	**0.001**			**0.001**			**0.001**		
**Religion**									
Hindu	440(34.8)	1,263	1.0	960(38.8)	2,474	1.0	975(44.0)	2,217	1.0
Budh/Muslim/Kirat/Christian	97(38.3)	253	1.2(0.9–1.5)	161(40.5)	398	1.1(0.86–1.3)	162(34.9)	464	0.7(0.6–0.9)
**P value**	**0.22**			**0.31**			**0.001**		
**Woman’s occupation**									
Not working outside home	30(51.7)	58	1.0	189(49.5)	382	1.0	500(46.7)	1,071	1.0
Skilled/unskilled working	62(46.6)	133	0.8(0.4–1.5)	151(52.6)	288	1.1(0.8–1.5)	167(44.4)	376	0.9(0.7–1.2)
Agriculture	445(33.6)	1,325	0.5(0.3–0.8)	781(35.5)	2,202	0.6(0.5–0.7)	470(38.1)	1,234	0.7(0.6–0.8)
**P value**	**0.001**			**0.001**			**0.001**		
**Husband’s occupation**									
Unskilled work	86(33.7)	255	1.0	187(32.9)	568	1.0	160(39.0)	409	1.0
Skilled work	251(39.8)	630	0.8(0.6–1.0)	601(45.2)	1,330	0.6(0.5–0.7)	747(45.7)	1,635	0.8(0.6–0.9)
Agriculture	200(31.7)	631	0.7(0.6–0.8)	333(34.2)	974	0.6(0.5–0.8)	230(36.1)	637	0.7(0.6–0.8)
**P-value**	**0.01**			**0.001**			**0.001**		
**Wealth quintile**									
Poor	391(31.4)	1,244	1.0	677(33.0)	2,053	1.0	318(33.3)	955	1.0
Middle	99(50.8)	195	2.3(1.7–3.0)	131(48.7)	269	2.0(1.5–2.5)	270(43.9)	615	1.6(1.3–1.9)
Rich	47(61.0)	77	3.0(2.0–5.5)	313(56.9)	550	2.7(2.2–3.3)	549(49.4)	1,111	2.0(1.6–2.3)
**P value**	**0.001**			**0.001**			**0.001**		
**Frequency to listening radio/reading newspaper/magazine**									
Not at all	88(28.7)	307	1.0	194(30)	646	1.0	257(34.1)	754	1.0
Less than once a week	178(32.6)	546	1.2(0.9–1.6)	403(37.6)	1,072	1.4(1.1–1.7)	494(41.5)	1,190	1.4(1.1–1.7)
At least once a week	271(40.9)	663	1.7(1.3–2.3)	524(45.4)	1,154	1.9(1.6–2.4)	386(52.4)	737	2.0(1.7–2.6)
**P-value**	**0.001**			**0.001**			**0.001**		
**Birth order**									
1st	161(30)	537	1.0	380(33.9)	1,121	1.0	400(35.2)	1,136	1.0
2nd	132(35.1)	576	1.3(0.9–1.7)	307(41.4)	742	1.4(1.1–1.7)	355(48.2)	737	1.7(1.4–2.0)
3rd+	244(40.5)	603	1.6(1.2–2.0)	434(43)	1,009	1.5(1.2–1.8)	382(47.3)	808	1.6(1.3–2.0)
**P value**	**0.001**			**0.001**			**0.001**		
**Child status**									
Alive	520(38.0)	1,367	1	1,088(41.7)	2,609	1.0	1,102(44.7)	2,466	1.0
Dead	17(11.4)	149	0.2(0.1–0.4)	33(12.5)	263	0.2(0.1–0.3)	35(16.3)	215	0.2(0.1–0.3)
**P-value**	**0.001**			**0.001**			**0.001**		

Woman’s education has an effect on the utilization of ANC services. When compared to women with no formal education, women with primary and secondary and above level education use these services by twice and more than thrice, respectively, across all the three zones. The proportion of women utilizing ANC services increases with the education level of women (*p* = 0.001). A higher level of husband’s education increases the utilization of ANC services across all zones (*p* = 0.001). Women whose husbands’ are educated utilize ANC services by one and a half, two and a half and over three and a half times more than those women whose husbands have had no formal education. Religion does not have a statistically significant role in utilization of ANC services in the Mountain and Hill zones. However, there is a significant difference between Budh/Muslim/Kirat/Christian and Hindus in the utilization of ANC services in the Terai zone (OR, 0.7; 95% CI [0.6–0.9]).

Women engaged in agriculture are at a significant disadvantage in utilizing ANC services compared to those not working outside home in all the three zones. Women whose husbands are engaged in agriculture suffer a similar disadvantage compared to those engaged in unskilled work in all zones. Skilled workers also show a statistical disadvantage in the Hill (OR, 0.6; 95% CI [0.5–0.7]) and Terai (OR, 0.8; 95% CI [0.6–0.9]) zones.

As wealth index increases, utilization of ANC services increases by twice for those in the middle and thrice for those in the rich and richer wealth quintiles in the Mountain and Hill zones. Utilization increases by one and half times (OR, 1.6; 95% CI [1.3–1.9]) and twice (OR, 2.0; 95% CI [1.6–2.3]) in the Terai zone with increase in wealth index. Across all zones, those listening to radio and/or reading newspapers and magazines at least once a week utilize ANC services by almost two times compared to those who do not listen to radio or read newspapers and magazines.

As birth order increases, women show greater awareness and utilize ANC services compared at first birth order across all zones. Women utilizing ANC services increase as the women experience pregnancies at higher birth order. Nearly 80% of those who report the status of child at birth as dead did not utilize ANC services across all zones.

### Safe delivery across the ecological zones

The total number of safe deliveries in Nepal in the three years prior to the survey is 1,814 that constitutes 25.7% of the total burden of deliveries in Nepal. These comprise of 245 births in the Mountain zone, 636 births in the Hill zone and 933 births in the Terai zone. Table 2 presents the results of univariate analyses of socio-economic and demographic characteristics of safe delivery according to ecological zones in Nepal. The table shows that as the age of the woman increases, chances of safe delivery decrease in the Mountain, Hill and Terai zones (*P* = 0.001). As compared with women in the 15–19 years, women above 30 years of age have less chances of safe delivery in the Mountain (OR, 0.07; 95% CI [0.04–0.1]), Hill (OR, 0.1; 95% CI [0.07–0.2]) and Terai (OR, 0.07; 95% CI [0.05–0.1]) zones. Women with place of residence in rural areas have less chance of safe delivery in the Mountain (OR, 0.3; 95% CI [0.17–0.5]), Hill (OR, 0.2; 95% CI [0.16–0.24]) and Terai (OR, 0.5; 95% CI [0.4–0.6]) zones, respectively.

**Table 2:** Socio-economic and demographic characteristics and univariate analyses of safe delivery services according to ecological zones in Nepal, NDHS-2011.

	Mountain zone			Hill zone			Terai zone		
Variable	Safe delivery 245(%)	Total 1,516	OR & 95%CI	Safe delivery 636(%)	Total 2,872	OR & 95%CI	Safe delivery 933(%)	Total 2,681	OR & 95%CI
**Age**									
15–19 yrs	30(44.8)	67	1.0	57(50.9)	112	1.0	115(73.7)	156	1.0
20–24 yrs	113(33.2)	340	0.6(0.4–1.1)	256(37.8)	677	0.6(0.4–0.9)	367(50.5)	727	0.4(0.3–0.5)
25–29 yrs	69(15.4)	448	0.2(0.1–0.4)	206(24.6)	838	0.3(0.2–0.5)	303(33.4)	908	0.2(0.1–0.3)
30+ yrs	33(5.0)	661	0.07(0.04–0.1)	117(9.4)	1,245	0.1(0.07–0.2)	148(16.6)	890	0.07(0.05–0.1)
**P value**	**0.001**			**0.001**			**0.001**		
**Place of residence**									
Urban	28(38.9)	71	1.0	216(51.7)	417	1.0	339(46.9)	723	1.0
Rural	217(15.0)	1,445	0.3(0.17–0.5)	420(17.1)	2,455	0.2(0.16–0.24)	594(30.3)	1,958	0.5(0.4–0.6)
**P value**	**0.001**			**0.001**			**0.001**		
**Woman’s education**									
No formal education	65(6.5)	1,004	1.0	133(8.7)	1,529	1.0	307(20.4)	1,506	1.0
Primary	69(28.2)	245	5.7(4.0–8.0)	113(20.4)	554	2.7(2.0–3.5)	169(38.6)	438	2.5(2.0–3.1)
Secondary & above	111(41.6)	267	10(7.0–1.5)	390(49.5)	789	10(8.0–13.0)	457(62)	737	6.4(5.0–7.7)
**P value**	**0.001**			**0.001**			**0.001**		
**Husband’s education**									
No formal education	21(5.0)	420	1.0	48(6.5)	735	1.0	127(15.8)	804	1.0
Primary	47(9.4)	500	2.0(1.2–3.4)	115(14.4)	796	2.4(1.7–3.4)	188(30.0)	626	2.3(1.8–3.0)
Secondary	141(29.5)	478	8.0(5.0–13.0)	335(30.8)	1,089	6.0(4.6–8.8)	465(45.1)	1,032	4.0(3.5–5.5)
Higher	36(30.5)	118	8.3(4.6–15.0)	138(54.8)	252	17(11.8–25.0)	153(69.9)	219	12.0(8.7–17.0)
**P value**	**0.001**			**0.001**			**0.001**		
**Religion**									
Hindu	204(16.2)	1,263	1.0	555(22.4)	2,474	1.0	819(36.9)	2,218	1.0
Budh/Muslim/Kirat/Christian	41(16.2)	253	0.9(0.7–1.2)	81(20.4)	398	0.9(0.7–1.2)	114(24.6)	463	0.6(0.4–0.7)
**P value**	**0.98**			**0.35**			**0.001**		
**Woman’s occupation**									
Not working outside home	29(50.0)	58	1.0	164(42.9)	380	1.0	473(44.2)	1071	1.0
Skilled/unskilled working	46(34.6)	132	0.5(0.3–1.0)	132(46)	285	1(0.8–1.5)	148(39.4)	376	0.8(0.6–1.0)
Agriculture	170(12.8)	1,326	0.15(0.1–0.3)	340(15.4)	2,207	0.24(0.2–0.3)	312(25.3)	1,234	0.43(0.4–0.5)
**P–value**	**0.001**			**0.001**			**0.001**		
**Husband’s occupation**									
Unskilled work	22(8.6)	255	1.0	79(13.9)	568	1.0	105(25.6)	412	1.0
Skilled work	161(25.5)	631	0.3(0.2–0.4)	434(32.6)	1,330	0.33(0.3–0.4)	677(41.5)	1,632	0.5(0.4–0.6)
Agriculture	62(9.8)	630	0.32(0.2–0.4)	123(12.6)	974	0.3(0.2–0.37)	151(23.7)	637	0.4(0.36–0.5)
**P-value**	**0.001**			**0.001**			**0.001**		
**Wealth quintile**									
Poor	132(10.6)	1,294	1.0	244(11.9)	2,051	1.0	182(19.1)	953	1.0
Middle	69(35.4)	195	4.6(3.0–6.5)	89(33.1)	270	3.7(2.8–4.9)	209(34.0)	615	2.0(1.7–2.8)
Rich	44(57.1)	77	11(7.0–18.0)	303(55.1)	551	9.0(7.0–11)	542(48.7)	1,113	4.0(3.6–4.9)
**P-value**	**0.001**			**0.001**			**0.001**		
**Frequency to listening radio/reading/newspaper/magazine**									
Not at all	26(8.5)	306	1.0	81(12.5)	648	1.0	173(22.9)	755	1.0
Less than once a week	76(13.9)	547	1.7(1.1–2.8)	183(17.1)	1,071	1.4(1.1–1.9)	399(33.5)	1,189	1.7(1.4–2.1)
At least once a week	143(21.6)	663	3.0(1.9–4.6)	372(32.3)	1,153	3.3(2.6–4.3)	361(49.0)	736	3.2(2.6–4.0)
**P-value**	**0.001**			**0.001**			**0.001**		
**Birth order**									
1st	122(22.7)	537	1.0	329(29.3)	1,123	1.0	460(40.5)	1,137	1.0
2nd	62(16.5)	376	0.7(0.5–0.9)	174(23.5)	741	0.7(0.6–0.9)	260(35.3)	737	0.8(0.7–0.9)
3rd+	61(10.1)	603	0.4(0.3–0.5)	133(13.2)	1,008	0.4(0.3–0.46)	213(26.4)	807	0.5(0.4–0.6)
**P-value**	**0.001**			**0.001**			**0.001**		
**Child status**									
Alive	231(16.9)	1,367	1.0	605(23.5)	2,609	1.0	894(36.3)	2,464	1.0
Dead	14(19.4)	149	0.5(0.3–0.9)	31(11.8)	263	0.4(0.3–0.7)	39(18.0)	217	0.4(0.3–0.6)
**P-value**	**0.02**			**0.001**			**0.001**		

Woman’s education has a significant effect on safe delivery. The chances of safe delivery for women with primary and secondary and above level education increase by two to ten times respectively, across all the three zones, when compared to women with no formal education. The difference is more pronounced in the Mountain and Hill zones than in the Terai zone. Higher level of Husband’s education also significantly increases safe delivery by two to seventeen times across all zones. Husbands who are educated to higher than secondary level ensure safe delivery by eight (OR, 8.3; 95% CI [4.6–15.0]), seventeen (OR, 17; 95% CI [11.8–25.0]) and twelve times (OR, 12; 95% CI [8.7–17.0]) compared to husbands who have had no formal education across the three zones. Religion does not have a statistically significant role in ensuring safe delivery in the Mountain and Hill zones, but there are less chances of safe delivery among the Budh/Muslim/Kirat/Christians than the Hindus in the Terai zone (OR, 0.6; 95% CI [0.4–0.7]).

Women who are either not working outside home or are in skilled or unskilled work have safe deliveries in the Hill and Terai zones but are at a disadvantage in the Mountain zone. Moreover, women engaged in agriculture are significantly disadvantaged in the Mountain (OR, 0.15; 95% CI [0.1–0.3]), Hill (OR, 0.24; 95% CI [0.2–0.3]) and Terai (OR, 0.43; 95% CI [0.4–0.5]) zones. Women whose husbands are engaged in agriculture or in skilled labour suffer a similar disadvantage compared to those engaged in unskilled work across all zones.

As wealth index increases, the number of safe deliveries increases in the Mountain, Hill and Terai zones. The difference is two (OR, 2.0; 95% CI [1.7–2.8]) and four times (OR, 4.0 ; 95% CI [3.6–4.9]) compared to the poor in the Terai zone, nearly four (OR, 3.7; 95% CI [2.8–4.9]) and nine times (OR, 9.0; 95% CI [7.0–11]) compared to the poor in the Hill zone and nearly five (OR, 4.6; 95% CI [3.0–6.5]) and eleven times (OR, 11.0; 95% CI [7.0–18.0]) compared with the poor in the Mountain zone. Those listening to radio and/or reading newspapers and magazines at least once a week are more likely to have safe deliveries by three times compared to those who do not. As birth order increases, women are disadvantaged by almost 50% in having safe deliveries when compared at first birth order across all zones. Nearly 50% of those who report the status of child at birth as dead did not have safe delivery across all zones.

### Univariate and multivariate analyses of combined data

The data was pooled together for multivariate analyses due to less number of women in segregated categories. Table 3 presents the univariate and multivariate findings of socio-economic and demographic predictors related to ANC and safe delivery services. Place of residence was a significant factor in univariate and multivariate analyses. Women residing in rural areas are less likely (OR 0.8, 95% CI [0.7–0.9]) to receive ANC than those living in urban areas. Moreover, women living in rural areas are less likely (OR, 0.6; 95% CI [0.5–0.7]) to have safe delivery compared to those in urban areas. While different ecological zones lose their significance in multivariate analyses for ANC, safe deliveries are significantly more in the Terai zone (OR, 1.7; 95% CI [1.2–2.1]). Women’s employment in agriculture and husband’s employment in skilled work and agriculture are significant only in the univariate analyses for ANC. An increase in wealth index ensures better utilization of ANC services and shows that safe deliveries increase by one and a half times for women in the middle (OR, 1.6; 95% CI [1.3–1.9]) and rich (OR, 1.8; 95% CI [1.5–2.2]) wealth index categories. Woman’s education has a comparable significant role both in utilization of ANC and safe delivery services. Husband’s education also plays an important role in utilization of ANC services and ensuring safe delivery. While religion plays little role in utilization of ANC services, Budh/Muslims/Kirat/Christians are at a disadvantage in ensuring safe deliveries (OR, 0.8; 95% CI [0.7–0.9]). Frequency to listening radio/reading newspaper/magazine for less than once a week and at least once a week are significant in univariate analyses for ANC. However, only listening/reading at least once a week is significant in multivariate analyses for ANC services. Birth order greater than one increases the odds of having ANC more than four to thirteen times in multivariate analyses.

**Table 3:** Univariate and multivariate analyses of socioeconomic and demographic predictors related to ANC and safe delivery services in Nepal.

Variable	Antenatal care		Safe delivery	
	Univariate	Multivariate	Univariate	Multivariate
	OR & 95% CI	OR & 95% CI	OR & 95% CI	OR & 95% CI
**Age**				
15–19 yrs	1	1	1	1
20–24 yrs	0.2(0.2–0.3)	0.1(0.09–0.2)	0.5(0.4–0.6)	0.4(0.3–0.6)
25–29 yrs	0.1(0.07–0.1)	0.03(0.02–0.1)	0.2(0.19–0.3)	0.2(0.1–0.2)
30+ yrs	0.04(0.03–0.1)	0.01(0.01–0.02)	0.08(0.06–0.1)	0.1(0.06–0.1)
**Place of residence**				
Urban	1	1	1	1
Rural	0.6(0.6–0.7)	0.8(0.7–0.9)	0.3(0.3–0.3)	0.6(0.5–0.7)
**Zones**				
Mountain	1	1	1	1
Hill	1.2(1.0–1.3)	1.2(0.9–1.4)	1.5(1.3–1.7)	1.2(0.9–1.4)
Terai	1.3(1.2–1.5)	1.1(0.9–1.5)	2.8(2.4–3.2)	1.7(1.2–2.1)
**Women’s education**				
No formal education	1	1	1	1
Primary	2.0(1.8–2.3)	1.5(1.3–1.8)	2.8(2.4–3.2)	1.7(1.4–2.0)
Secondary & above	3.6(3.2–4.1)	2.9(2.4–3.4)	8.0(7.0–9.0)	2.9(2.4–3.5)
**Husband education**				
No formal education	1	1	1	1
Primary	1.5(1.3–1.7)	1.2(1.03–1.4)	2.0(1.7–2.4)	1.3(1.04–1.6)
Secondary	2.6(2.3–3.0)	1.7(1.4–2.0)	5.1(4.3–6.04)	1.7(1.4–2.1)
Higher	4.1(3.4–5.0)	2.5(1.9–3.3)	11.2(9.0–13.9)	2.3(1.72–3.1)
**Religion**				
Hindu	1	1	1	1
Budh/Muslim/Kirat/Christian	0.9(0.8–1.1)	1.1(0.1–1.2)	0.7(0.6–0.9)	0.8(0.7–0.9)
**Woman’s occupation**				
Not working outside home	1	1	1	1
Skilled/unskilled working	1.1(0.9–1.2)	1.2(0.9–1.5)	0.9(0.7–1.1)	0.9(0.8–1.2)
Agriculture	0.6(0.5–0.7)	1.0(0.9–1.2)	0.3(0.2–0.3)	0.6(0.6–0.8)
**Husband’s occupation**				
Unskilled work	1	1	1	1
Skilled work	0.7(0.6–0.8)	1.0(0.9–1.3)	0.4(0.3–0.4)	0.8(0.7–0.9)
Agriculture	0.6(0.6–0.7)	1.0(0.9–1.2)	0.3(0.3–0.4)	0.7(0.6–0.9)
**Wealth quintile**				
Poor	1	1	1	1
Middle	1.8(1.6–2.1)	1.3(1.1–1.6)	3.4(2.9–4.0)	1.6(1.3–1.9)
Rich	1.6(2.0–2.5)	1.4(1.2–1.7)	7.0(6.1–7.9)	1.8(1.5–2.2)
**Frequency to listening radio/reading newspaper/magazine**				
Not at all	1	1	1	1
Less than once a week	1.3(1.2–1.5)	1.1(0.9–1.3)	1.6(1.3–1.8)	1.1(0.9–1.3)
At least once a week	1.9(1.6–2.1)	1.2(1.0–1.5)	2.7(2.3–3.1)	1.4(1.1–1.7)
**Birth order**				
1st	1	1	1	1
2nd	1.5(1.3–1.7)	3.8(3.2–4.4)	0.8(0.7–0.9)	1.3(1.1–1.5)
3rd+	1.5(1.4–1.7)	13.6(11.3–16.5)	0.4(0.4–0.5)	2.0(1.7–2.5)
**Child status**				
Alive	1	1	1	1
Dead	0.2(0.2–0.3)	0.2(0.2–0.3)	0.4(0.3–0.5)	0.6(0.5–0.8)

**Figure 1:**
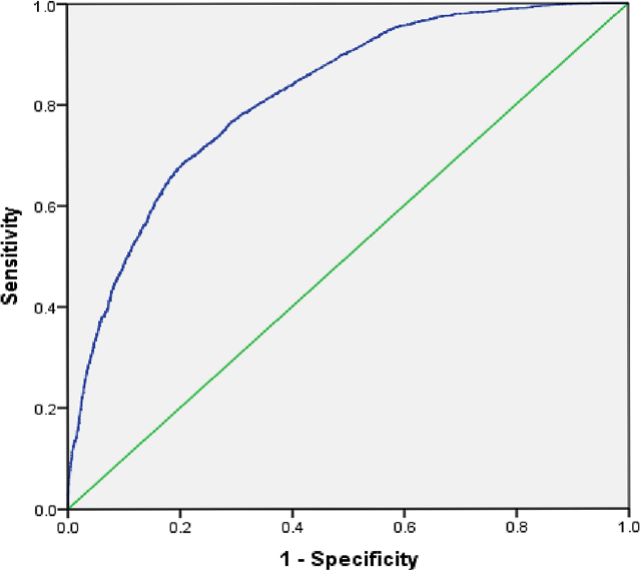
Receiver operating characteristic (ROC) curve for predicting utilization of ANC services. Area under the curve (ROC) = 0.82, 95% CI [0.81–0.83].

**Figure 2:**
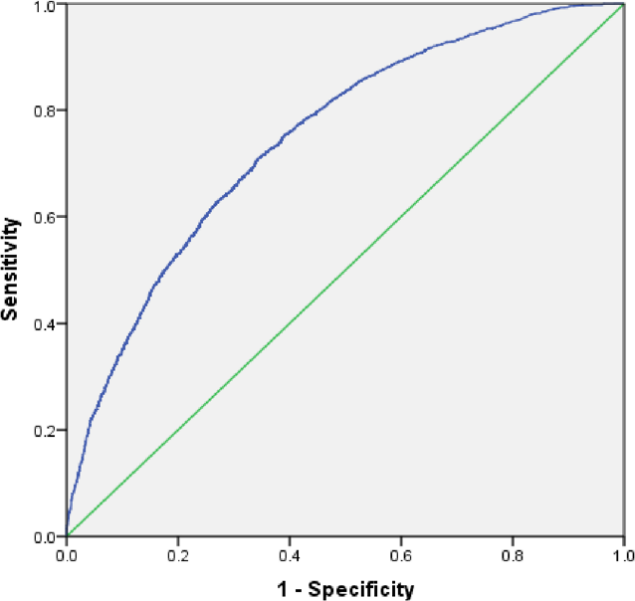
Receiver operating characteristic (ROC) curve for predicting utilization of safe delivery services. Area under the curve (ROC) = 0.75, 95% CI [0.74–0.76].

The AUROC (Fig. 1) shows that the model for utilization of ANC services is able to discriminate between women who received ANC and those who did not with 82% accuracy (95% CI [0.81–0.83]). The AUROC (Fig. 2) shows that the predicted model for safe delivery services is able to discriminate between women who have safe delivery and those who do not with 75% accuracy (95% CI [0.74–0.76]). These values indicate that the developed models are fair enough for appropriate prediction of ANC and safe delivery services.

## Discussion

The present study shows socio-economic and demographic factors associated with ANC and safe delivery services across the three ecological zones in Nepal. Though the two outcome variables, ANC services and safe delivery are analysed separately, there is evidence to support that women who access ANC are more likely to have a SBA at delivery. Young women who initiated ANC early are more likely to use skilled professional assistance at delivery (Ochako et al., 2011; Gabrysch & Campbell, 2009; Paul & Rumsey, 2002; Gage & Guirlène, 2006; Anwar et al., 2004; Islam, Islam & Yoshimura, 2014). It is projected that higher utilization of ANC services increases perinatal survival. However, frequently documented deterrents to utilization of ANC are: women have no time, are in good health, are embarrassed or live far away (Yang et al., 2010). ANC could also be a full day’s investment that discourages attendance (Andrew et al., 2014). This study was conducted to enhance knowledge about the increased use of ANC and safe delivery services in Nepal.

Disaggregation of data, according to ecological zones, highlights socio-economic and demographic imbalances in accessing ANC services and ensuring safe delivery. The WHO highlights the lack of access to local, adequately resourced health care facilities as an important reason for the slow progress towards achieving MDG-5 goals (Gupta et al., 2014; Finlayson & Downe, 2013). Often women have only a vague understanding of specific ANC procedures (Pell et al., 2013). In Tanzania, 96% pregnant women attended at least one ANC with a skilled worker (Gupta et al., 2014). In Bangladesh, about 50% pregnant women received at least one ANC visit (NIPORT, 2009). 94% pregnant women in Zambia attended ANC at least once with a skilled provider, while 74% attended the recommended four antenatal visits (Kyei, Campbell & Gabrysch, 2012; CSO , 2009; WHO, 2010). However, maternal mortality rates are high in each of these countries. Fewer than 40% women received any ANC from a trained provider, and fewer than 10% births took place in a health facility in Nepal in 2001 (Furuta & Salway, 2006). These figures are important because they are a pointer to under-utilization of ANC and safe delivery services.

### Univariate analyses of ANC services across ecological zones

In the NDHS-2001 survey, 65% women received ANC in the Terai zone while only 23% received ANC in the Hill zone (NDHS 2001). Overall, in the 2011 survey, though 80% women utilized ANC services in the Terai zone, only 50% received ANC services in Hill and Mountain zones. This is an improvement over the NDHS-2001 figures but points to further need of improvement in outreach of ANC services. This finding is similar to various other studies that have found regional imbalances in Kenya (Magadi, Madise & Rodrigues, 2000) and Guatemala (Glei, Goldman & Rodríguez, 2003).

Univariate analyses of socio-economic and demographic factors affecting utilization of ANC services across the ecological zones reveal that age, place of residence, education, occupation, religion, wealth index, frequency of listening to radio/reading newspapers and birth order are statistically significant. As age increases, the proportion of women utilizing ANC services decreases. This may be due to the fact that the woman has already had previous pregnancies and therefore is reticent in utilizing ANC services. As compared to women in the urban areas, only half the women in the rural areas access ANC services across all ecological zones. Women in the Terai zone are at a disadvantage; however, the disadvantage is even greater for women in the Mountain and Hill zones. Women with primary and secondary education are approximately twice and thrice more likely to access ANC than women with no formal education across all zones. Husband’s education also shares a positive relationship in accessing ANC services. Middle and Rich wealth groups significantly increase the chances of utilization of ANC services compared to the poor. Public messaging is important on radio and television. 33.3% women listened to radio and 16.8% watched TV at least weekly (MoHP, New ERA and ORC Macro, 2002). This kind of exposure to public messaging helps in bettering utilization of ANC services by almost two times in the Mountain, Hill and Terai zones. Higher birth order is inversely and significantly associated with the number of ANC visits by the expecting mother. This is detrimental to safe motherhood since the risk associated with childbirth increases with age and could adversely affect the health of the mother (Nketiah-Amponsah, Senadza & Arthur, 2013). However, women at higher birth order are more likely to utilize full ANC services than women at lower birth order. Our study shows that though older women are less likely to opt for ANC services, these women are also more likely to utilize complete ANC services at higher birth order.

### Univariate analyses of safe delivery services across ecological zones

In the previous survey, 12.1% of women under age 20 gave birth in health facilities compared with 8.9% of women aged 20–34 and 3.6% of women over 35 (MoHP, New ERA and ORC Macro, 2002). Nationally, the percentage of women who delivered with assistance from a SBA is low at 36% in 2011 (MoHP, New ERA, and ICF International Inc, 2012). In this survey, the overall percentage of women who gave birth in a health facility had increased by almost double. However, rural place of residence is significantly disadvantaged in ensuring safe delivery. Primary and secondary education of the woman, husband’s education and wealth index are significantly advantaged in ensuring safe delivery. Exposure to public messaging increases safe deliveries by three times in the Mountain, Hill and Terai zones.

### Univariate and multivariate analyses of utilization of ANC and safe delivery services in Nepal

In our study, the ecological zones lose their significance in the multivariate analyses for ANC services. This is similar to another study on Nepal (Joshi et al., 2014). However, safe delivery remains a major challenge in ensuring mother and child survival in the Hill and Mountain zones with safe deliveries being significantly more in the Terai zone. Place of residence is a significant factor in univariate and multivariate analyses of combined data. This is supported by several other studies that state that women in urban areas are more likely than women in rural areas to use ANC services in Jordan (Obermeyer & Potter, 1991), Guatemala (Pebley, Goldman & Rodriguez, 1996) and Thailand (Raghupathy, 1996). However, place of residence was not a significant factor in the zone-wise analyses in the Terai zone. This shows that rural place of residence has differences depending on the zones. Women in the rural areas of the hill and the mountain zones are more severely disadvantaged than in the Terai zone. Primary and secondary education of the woman, husband’s education and increasing wealth index are significant factors in multivariate analyses. Middle and Rich wealth groups significantly increase utilization of ANC and safe delivery services compared to the poor. Other studies on Nepal and elsewhere have recommended that woman’s education along with wealth indicators remain the greatest challenges to utilization of ANC services (Joshi et al., 2014; Osorio, Tovar & Rathmann, 2014). Overall, Budh/Muslim/Kirat/Christians are not at a significant disadvantage in accessing ANC, though they are at a disadvantage in the Terai zone in the zone-wise analyses. However, Budh/Muslim/Kirat/Christians are significantly disadvantaged in ensuring safe deliveries compared to Hindus in univariate and multivariate analyses of combined data. While Budh/Muslim/Kirat/Christians were significantly disadvantaged in the Terai zone in zone-wise analyses of safe delivery services, religion had no significance in the mountain and hill zones. This factor needs further investigation for appropriate programmes designed to understand the needs of these religious communities, especially in the Terai zone. Husband’s occupation in the skilled sector was not a significant factor in the mountains in the zone-wise analyses but was significant in the univariate analyses of combined data. However, this was not a significant factor in multivariate analyses of ANC services. This factor also needs investigation for any definitive recognition of its influence on use of ANC services.

The AUROC is used to see if the model fits the data. The prediction models demonstrate discrimination with a value of 0.82 for ANC services and 0.75 for safe delivery. This establishes that the developed models reliably predict the association between outcome and independent variables.

## Limitations

The data were self-reported and the retrospective nature of the study allows the data to be subject to recall bias. The study tried to minimise the bias by selecting women who had had a birth in the three years preceding the survey. However, most of the variables (age, wealth index, education, occupation) are recorded at the time of the survey rather than at the time of birth of the child. These variables may have changed since the birth of the child. Thirdly, our study did not analyse caste distinctions because caste is restricted to the Hindu religion. However, since Nepal is a predominantly Hindu country, it would be worthwhile to distinguish caste-based inequalities in utilization of ANC and safe delivery services. A study revealed that the more privileged Newar and Hill Brahman women have the highest percentage of delivery in a health facility supported by a SBA, while Terai/Madhesi Dalit have the lowest levels for both services (Pandey et al., 2013). Fourthly, it is often not the lack of facility or the access to facility but the quality of service that hampers utilization of ANC services. Our study did not analyse the quality of services.

## Conclusion

We surmise that ANC services have increased substantially from the last NDHS survey. Poor utilization of services is related to a complex set of social and demographic factors that affect the use, accessibility, affordability and perception about the need and utility of such services (WHO, 2012a; WHO, 2012b; Paxton & Wardlaw, 2011). While being poor with no formal education of self and husband living in a rural area were a deterrent to accessing ANC services, the odds of having a safe delivery were significantly more in the Terai zone. There is definitive need to emphasise safe delivery and to ensure that women in the Hill and Mountain zones have access to safe delivery. It means that the areas covered by SBAs are increased and special incentives are offered to train women social workers in the Hill and Mountains.

The study recommends that disaggregated targets, according to ecological zones, be set so as to further reduce maternal mortality rates in Nepal. Further, Nepal runs an Aama program that comprises free delivery services and cash incentives to cover travel costs for normal delivery, management of complications, and caesarean section (Shreshtha, 2013). This initiative needs a detailed audit to provide insights into how the ANC and safe delivery services may be further enhanced.

## Additional Information and Declarations

### Data Availability

The following information was supplied regarding the availability of data: Data is available on submission and approval for a proposal of study from the DHS website: http://dhsprogram.com/data/new-user-registration.cfm.
